# Modest dose anti-thymocyte globulin administered intraoperatively is safe and effective in kidney transplantations: a retrospective study

**DOI:** 10.7717/peerj.7274

**Published:** 2019-08-16

**Authors:** Hui-Ying Liu, Yuan-Tso Cheng, Hao Lun Luo, Chiang-Chi Huang, Chien Hsu Chen, Yuan-Chi Shen, Wen-Chin Lee

**Affiliations:** 1 Department of Urology, Kaohsiung Chang Gung Memorial Hospital and Chang Gung University College of Medicine, Kaohsiung, Taiwan; 2 Division of Nephrology, Department of Internal Medicine, Kaohsiung Chang Gung Memorial Hospital and Chang Gung University College of Medicine, Kaohsiung, Taiwan

**Keywords:** Anti-thymocyte globulin, Induction, Kidney transplantation

## Abstract

**Background:**

Anti-thymocyte globulin (ATG) as induction therapy in renal transplantation is facing the dilemma of reducing the incidence of acute rejection (AR) and delayed graft function (DGF) or increasing risks of infection and malignancy. The purpose of this study was to delineate the safety and efficiency of the optimal ATG dosage.

**Methods:**

We retrospectively evaluated 91 deceased donor kidney transplant recipients (KTRs) in our institution between March 2011 and January 2019. The patients were classified into three groups based on induction therapy: (1) Group 1: modest-dose ATG (three mg/kg) intraoperatively (*N* = 21); (2) Group 2: low-dose ATG (1–1.5 mg/kg) intraoperatively (*N* = 23); (3) Group 3: basiliximab 20 mg both on day 0 and 4 (*N* = 47). In Groups 1 and 2, all patients received a daily low-dose program (1–1.5 mg/kg each day) with target dosage of six mg/kg. Induction therapy was combined with standard immunosuppressive regimen consisting of calcineurin inhibitors, mycophenolate/the mammalian target of rapamycin inhibitors and corticosteroids.

**Results:**

There was no significant difference in patient characteristics among groups. The outcomes of infection rate, biopsy-proven acute rejection, post-transplant diabetes mellitus, graft survival, and patient survival were similar among groups. Compared to the daily low-dose ATG regimen, the intraoperative modest-dose regimen did not cause more dose interruption and hence was more likely to reach the target ATG dosage. The intraoperative modest-dose regimen also seemed to reduce the rate of DGF.

**Discussion:**

In recent years, a trend of using a “lower” dose of ATG has seemed to emerge. Our results suggest intraoperative modest-dose ATG followed by daily low-dose ATG regimen was safe and effective in cadaveric renal transplantations for preventing DGF, AR, and graft loss.

## Introduction

Anti-thymocyte globulin (ATG) induction therapy is known to reduce the incidence of acute rejection (AR), delayed graft function (DGF), and graft loss in renal transplantation (Ciancio, Burke & Miller, 2007; Erickson et al., 2010; Kainz et al., 2010; Knoll, 2008; Mourad et al., 2012; Schenker et al., 2011; Siedlecki, Irish & Brennan, 2011; Thiyagarajan, Ponnuswamy & Bagul, 2013). It is recommended for recipients with moderate to high risk of AR and DGF (Bia et al., 2010; Kasiske et al., 2009), though the risk of infection and malignancy might be increased (Clesca et al., 2007; Laftavi et al., 2011; Meier-Kriesche, Arndorfer & Kaplan, 2002; Mourad et al., 2012; Schenker et al., 2011). Currently, the timing of administration and the ideal dosage of ATG induction therapy remain inconclusive as most clinical studies have been carried out on patients with different immunological risk and in the context of varying maintenance regimens (Mourad et al., 2012; Schenker et al., 2011). Nevertheless, in recent years, a trend of using a “lower” dose of ATG has seemed to emerge. The purpose of this study was to examine the safety and efficiency of intraoperative modest-dose ATG followed by daily low-dose ATG regimen in cadaveric renal transplantations.

## Patients and methods

We retrospectively evaluated 155 adult kidney transplant recipients (KTRs) who received either standard or expanded criteria deceased donor in our institution from March 2011 to January 2019. Patients were excluded because of not-receiving induction therapy (*N* = 10), or receiving living donor kidney transplantation (*N* = 54). A total of 91 patients were enrolled in our study.

**Figure 1:**
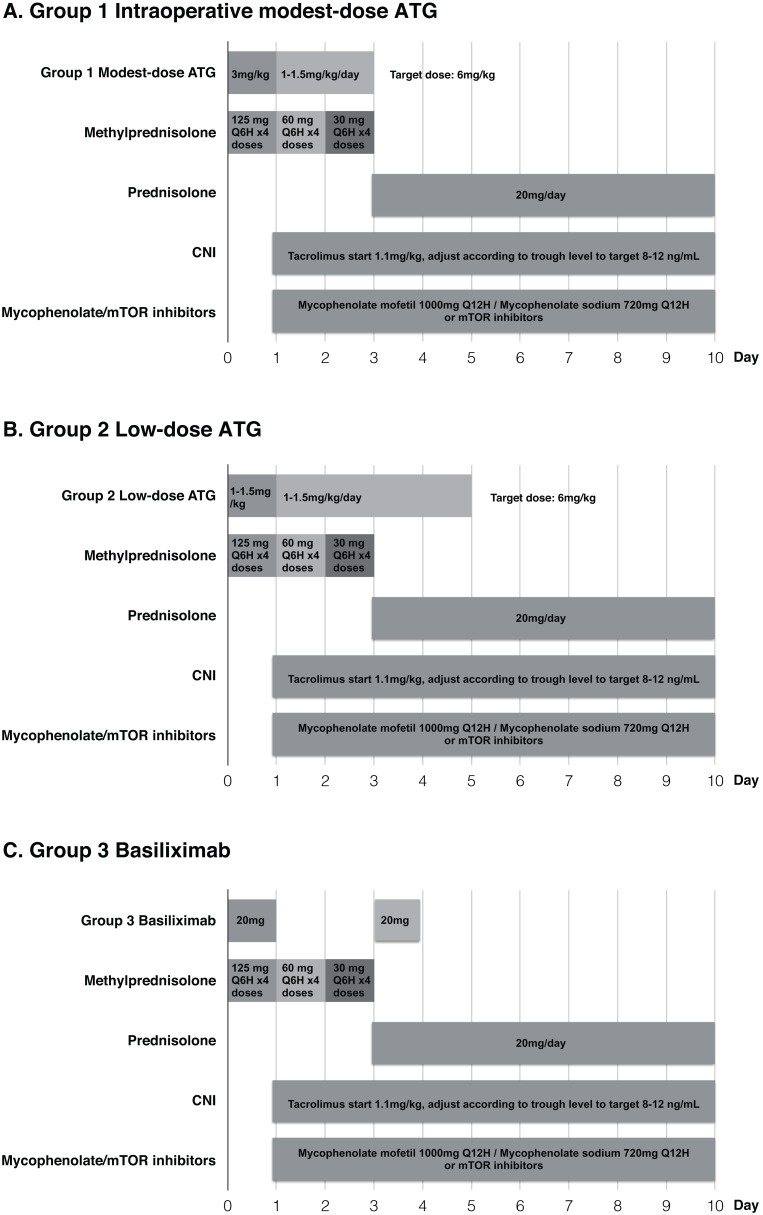
Immunosuppressive regimens in the three groups. (A) Group 1 Intraoperative modest-dose ATG, (B) Group 2 Low-dose ATG, (C) Group 3 Basiliximab.

Based on the recommendations from kidney disease: improving global outcomes (KDIGO) guidelines (Kasiske et al., 2009), we used rabbit ATG (Thymoglobuline^®^, Genzyme) induction therapy in intermediate to high risk patients and chose basiliximab for low immunologic risk recipients. According to the guidelines, low immunologic risk was defined as first-time transplant recipients who have less than 20% panel-reactive antibodies. Intermediate risk was defined as transplant recipients with panel-reactive antibodies between 20% and 80%. All the enrolled patients were classified into three groups: (1) Group 1: Recipients were administered modest-dose ATG (three mg/kg) intraoperatively after patient was anesthetized in the operation room (*N* = 21); (2) Group 2: Recipients were administered low-dose ATG (1–1.5 mg/kg) intraoperatively (*N* = 23); (3) Group 3: Patients received basiliximab with 20 mg dose on day 0 and 4 (*N* = 47). In Groups 1 and 2, all patients received a daily low-dose program (1–1.5 mg/kg each day) with target dosage of six mg/kg. Standard maintenance immunosuppressive regimen consisting of calcineurin inhibitors, mycophenolate/the mammalian target of rapamycin inhibitors and corticosteroid was used in the three groups (Fig. 1). This study was approved by the Institutional Review Board of The Kaohsiung Chang Gung Memorial Hospital (Approval number: 201801012B0).

Baseline donor and recipient characteristics, operative variables, and post-transplantation outcomes including incidence of DGF, infection, malignancy, biopsy-proven acute rejection (BPAR), post-transplant diabetes mellitus (PTDM), graft survival and patient survival were evaluated during the first post transplantation year. Renal biopsies were performed based on the recommendation of KDIGO guidelines (Kasiske et al., 2009). IBM^®^ SPSS^®^ Statistics Base Version 24 software was used for all statistical analyses. Arithmetic values were calculated and expressed as mean ± SD. Baseline demographic data among patient groups were analyzed using one-way ANOVA for continuous variables and Pearson Chi-square test for dichotomous variables. Two-sided *p-*values less than 0.05 were considered to indicate statistical significance.

## Results

Among the three study groups, there were no significant differences in general characteristics of both donor and recipient including gender, age, body mass index, comorbidities (e.g., hypertension, diabetes mellitus, coronary artery disease, cerebral vascular accident), preoperative dialysis duration, warm ischemia time, kidney donor profile index, and kidney donor risk index (Table 1). In Group 1, 21 patients received intraoperative modest-dose ATG (three mg/kg). The mean administered cumulative ATG dosage was 4.54 ± 1.43 mg/kg. In Group 2, 23 patients received low dose ATG (1–1.5 mg/kg) intraoperatively, and their mean administered cumulative ATG dosage was 4.04 ± 1.78 mg/kg. The ATG therapy was held or ceased if leukocytopenia (WBC < 2,500/uL), thrombocytopenia (platelet < 100,000/uL), or high-grade fever episode occurred (>38.5 °C). The trend of ATG dose interruption or premature termination occurred less in Group 1 (14.3%) than Group 2 (26.1%) without significant difference. In Group 3, all patients receiving basiliximab completed the two doses on day 0 and day 4 as suggested.

**Table 1:** Recipient and donor characteristics.

	Group 1 intraoperative modest-dose ATG (*N* = 21)	Group 2 low-dose ATG (*N* = 23)	Group 3 basiliximab (*N* = 47)	*p*-value
Total ATG dosage (mg/kg)	4.54 ± 1.43	4.04 ± 1.78	0.62 ± 0.14	<0.001* (Group 1 = 2 > 3)
ATG dose interruption, *N* (%)	3 (14.3%)	6 (26.1%)	0 (0%)	0.004* (Group 2 > 3)
Gender (male)	8 (38.1%)	11 (47.8%)	22 (46.8%)	0.763
Age (year)	43.82 ± 17.28	49.47 ± 10.11	42.00 ± 11.05	0.068
BMI	22.33 ± 4.11	23.20 ± 3.58	23.52 ± 3.97	0.525
HTN, *N* (%)	15 (71.4%)	12 (52.2%)	30 (63.8%)	0.407
DM, *N* (%)	1 (4.8%)	1 (4.3%)	4 (8.5%)	>0.99
CAD, *N* (%)	0 (0%)	1 (4.3%)	1 (2.1%)	>0.99
CVA, *N* (%)	0 (0%)	1 (4.3%)	0 (0%)	0.484
Dialysis duration (year)	7.43 ± 4.84	10.45 ± 5.61	7.37 ± 5.15	0.061
OP time (min)	332.81 ± 67.88	323.30 ± 67.49	292.49 ± 41.65	0.011* (Group 1 > 3)
Warm ischemia time (min)	49.72 ± 13.01	45.25 ± 10.43	45.38 ± 9.32	0.314
KDPI (%)	59.30 ± 26.17	48.11 ± 26.30	44.98 ± 28.89	0.168
KDRI	1.16 ± 0.33	1.08 ± 0.47	1.01 ± 0.34	0.347

**Note:**

BMI, body mass index; HTN, hypertension; DM, diabetes mellitus; CAD, coronary artery disease; CVA, cerebral vascular accident; OP, operation; KDPI, kidney donor profile index; KDRI, kidney donor risk index. Data was expressed as absolute and relative frequencies or mean ± SD.

* The *p* value < 0.05 was regarded as statistically significant.

There were no significant differences in all-cause infection, cytomegalovirus infection, BK virus infection, urinary tract infection, BPAR, PTDM, graft survival, and patient survival (Table 2).

**Table 2:** Clinical outcomes of the three groups.

	Group 1 intraoperative modest-dose ATG (*N* = 21)	Group 2 low-dose ATG (*N* = 23)	Group 3 basiliximab (*N* = 47)	*p*-value
Follow-up, month	24.14 ± 20.32	54.78 ± 16.09	41.40 ± 22.90	<0.001* (Group 2 > 3 > 1)
DGF	9 (42.9%)	14 (60.9%)	8 (17.0%)	0.001* (Group 2 > 3)
All cause infection	11 (52.4%)	12 (52.2%)	25 (53.2%)	0.996
CMV infection	2 (10.0%)	1 (4.5%)	2 (4.7%)	0.707
BKV infection	3 (15.8%)	2 (9.1%)	8 (19.0%)	0.672
UTI	8 (38.1%)	6 (26.1%)	17 (36.2%)	0.639
Malignancy	0 (0%)	4 (17.4%)	3 (6.4%)	0.090
BPAR	5 (23.8%)	1 (4.3%)	7 (15.6%)	0.182
PTDM	2 (9.5%)	2 (8.7%)	2 (4.3%)	0.624
Hospitalization days	19.38 ± 13.98	21.48 ± 13.24	18.72 ± 17.33	0.786
Graft survival (1 year)	21 (100.0%)	22 (95.7%)	46 (97.9%)	>0.99
Re-transplant	0 (0%)	0 (0%)	0 (0%)	–
Patient survival (1 year)	19 (90.5%)	23 (100.0%)	47 (100.0%)	0.051

**Note:**

OP, operation; DGF, delayed graft function; CMV, cytomegalovirus; BKV, BK virus; UTI, urinary tract infection; BPAR, biopsy-proven acute rejection; PTDM, post-transplant diabetes mellitus.

* The *p* value < 0.05 was regarded as statistically significant.

We used the definition of requirement of dialysis in the first week following transplantation as DGF (Thiyagarajan, Ponnuswamy & Bagul, 2013). In our study, 31 patients were recorded as DGF after renal transplantation of which nine (42.9%) patients received intraoperative modest-dose ATG (Group 1), 14 (60.9%) patients received low-dose ATG (Group 2), and eight (17.0%) patients received basiliximab (Group 3). Compared to daily low dose ATG regimen, intraoperative modest-dose ATG induction seemed to offer less DGF, though the difference was not statistically significant.

## Discussion

Anti-thymocyte globulin induction therapy in kidney transplantation is suggested by most transplant guidelines, though the optimal dosage and administered timing are yet to be determined. Kaden et al. (1995, 2011) found a single high-dose bolus ATG given (nine mg/kg body weight) in reperfusion period could effectively improve kidney graft survival (Du et al., 2016; Kaden, Strobelt & May, 1998). Studies using single high-dose ATG were reported on efficacy in immunosuppression (Kaden et al., 2009; Samsel et al., 2008; Yussim & Shapira, 2000). However, full-dose ATG induction therapy (7–10 mg/kg) has been associated with increased infectious complications and morbidity in the early post-transplant period (Clesca et al., 2007; Laftavi et al., 2011). In recent years, a trend of using “reduced” dose of ATG has emerged (Gurk-Turner et al., 2008; Hardinger et al., 2005; Laftavi et al., 2011; Wong et al., 2006). Hardinger et al. (2005) showed the efficacy of reduced dose of ATG induction (modest-dose ATG (three mg/kg) intra-operatively followed by 1.5 mg/kg on POD 1 and 2) in 40 low-risk renal transplant recipients. It has not yet been tested whether the initial dose can be further reduced. In our study, we show that the initial low dose (1.5 mg/kg) regimen could be safe because BPAR, graft survival, and patient survival characteristics did not differ between Groups 1 and 2.

The mean total dose of ATG was comparable in both Groups 1 (4.54 ± 1.43 mg/kg) and 2 (4.04 ± 1.78 mg/kg). Gurk-Turner et al. (2008) compared the incidence of BPAR during the first 12 months and the graft survival in KTRs treated with ≤7.5 mg/kg or >7.5 mg/kg ATG. They concluded that in high risk KTRs, total ATG doses ≤7.5 mg/kg are safe and effective in achieving a low rate of AR and graft outcomes comparable to higher doses. In line with our results, Klem et al. examined the 1-year AR rate, patient survival and graft survival in KTRs receiving a total 4.5 mg/kg or six mg/kg of ATG. They reported 1-year AR rates were 10% and 11% in the 4.5 mg/kg and six mg/kg cohorts, respectively, with 100% patient and graft survival at 1 year in both groups (Klem et al., 2009). In addition, compared to Group 2, Group 1 regimen did not result in more dose interruption and hence was more likely to reach the target ATG dosage.

Notably, however, Group 1 in our study did show a lower DGF rate (42.9% vs. 60.9%). DGF is a major obstacle for long-term graft survival. Data from a prospective randomized clinical trial highlights the crucial role of intraoperative administration of ATG in reducing DGF (Goggins et al., 2003). This benefit disappeared when the initial dosing of ATG was reduced to as low as 1.5 mg/kg (Brennan et al., 2006). Based on these reported findings and our results, we would suggest initial modest-dose ATG is a more ideal induction regimen. By reducing DGF, the superiority of this initial modest-dose ATG induction regimen might be reflected in better long-term graft survival. Group 3 showed a significantly low DGF rate because this regimen, by guideline recommendations, was applied to the low immunologic risk recipients.

The risk of cancer, especially lymphoproliferative diseases, is one of the known side effects of anti-thymocyte induction agents (Schenker et al., 2011). Higher incidence of non-Hodgkin's lymphoma connected to antilymphocyte antibody induction has been previously described (Cockfield et al., 1991; Hibberd et al., 1999; Opelz & Henderson, 1993; Samsel et al., 2008). Nevertheless, in our study, the incidence of onset malignancy had no significant difference among the three groups. Although our study showed both modest-dose and low-dose ATG induction yielded comparable malignancy rates to basiliximab, longer follow-up periods are required to confirm this.

The main limitations of the current study were the small sample size and the unequal patient number in study groups. Although this phenomenon did truly reflect real world experience from a non-high volume kidney transplantation center, results from analysis on these patients may limit its generalizability. Further prospective studies with larger numbers of patients are required to confirm these results.

## Conclusions

We proposed a safe and effective ATG induction regimen in intermediate to high risk renal transplant recipients. In line with the trend of administering lower ATG dose in recent years, our dosing regimen not only confirmed the advantage of modest reduction of initial ATG dose to three mg/kg, but also demonstrated its potential benefits in less dose interruption and reducing DGF.

## Supplemental Information

10.7717/peerj.7274/supp-1 Supplemental Information 1 Renal function of the three study groups. The renal function was analyzed with one-way ANOVA and Scheffe post hoc test. To compare renal function outcomes with estimated glomerular filtration rate (mL/min/1.73m^2^) and creatinine level (mg/dL) during the postoperative follow-up period, there are no differences among three groups.

10.7717/peerj.7274/supp-2 Supplemental Information 2 Renal transplant raw data. Raw data was provided for analysis in this study.

## Additional Information and Declarations

### Data Availability

The following information was supplied regarding data availability: The raw data are available in the Supplemental Files.
