# Supplementary material for: Modest dose anti-thymocyte globulin administered intraoperatively is safe and effective in kidney transplantations: a retrospective study
Source: PeerJ. 2019 Aug 16;7:e7274. doi: 10.7717/peerj.7274 (PMC6699478; doi:10.7717/peerj.7274)
Supplement: Supplemental Information 1 — The renal function was analyzed with one-way ANOVA and Scheffe post hoc test. To compare renal function outcomes with estimated glomerular filtration rate (mL/min/1.73m2) and creatinine level (mg/dL) during the postoperative follow-up period, there are no differences among three groups. [file peerj-07-7274-s001.docx]

The renal function was analyzed with one-way ANOVA and Scheffe post hoc test. To compare renal function outcomes with estimated glomerular filtration rate (mL/min/1.73m^2^) and creatinine level (mg/dL) during the postoperative follow-up period, there are no differences among three groups.

Renal function of the three study groups

|  | **Group 1**  **Intraoperative modest-dose ATG (N=8)** | **Group 2**  **Low-dose ATG**  **(N=22)** | **Group 3**  **Basiliximab**  **(N=34)** | **p value** |
| --- | --- | --- | --- | --- |
| **Creatinine level (mg/dL)** | | | | |
| **Baseline** | 7.30±3.16 | 10.14±4.07 | 9.25±2.90 | 0.078 |
| **Postoperative Day 1** | 6.91±2.29 | 8.46±3.66 | 7.93±2.67 | 0.374 |
| **Postoperative Day 3** | 6.41±2.34 | 7.68±4.23 | 5.05±4.21 | 0.213 |
| **Postoperative Day 7** | 3.88±1.96 | 5.34±3.69 | 3.03±2.68 | 0.117 |
| **Postoperative Month 1** | 1.60±0.46 | 1.59±0.89 | 1.50±0.73 | 0.937 |
| **Postoperative Month 3** | 1.47±0.41 | 1.68±1.89 | 1.36±0.44 | 0.747 |
| **Postoperative Month 6** | 1.42±0.54 | 1.66±1.82 | 1.34±0.48 | 0.745 |
| **Postoperative Month 12** | 1.68±1.14 | 1.80±2.38 | 1.37±0.85 | 0.808 |
| **Estimated glomerular filtration rate (mL/min/1.73m^2^)** | | | | |
| **Baseline** | 9.47±7.24 | 6.05±2.79 | 6.45±2.30 | 0.059 |
| **Postoperative Day 1** | 9.03±5.33 | 7.08±2.21 | 7.70±2.55 | 0.196 |
| **Postoperative Day 3** | 12.40±14.32 | 9.79±7.18 | 27.58±26.87 | 0.072 |
| **Postoperative Day 7** | 24.07±25.37 | 19.44±17.70 | 40.84±28.92 | 0.075 |
| **Postoperative Month 1** | 46.16±21.37 | 51.26±17.80 | 54.83±19.67 | 0.472 |
| **Postoperative Month 3** | 49.99±19.54 | 58.10±23.56 | 59.28±20.81 | 0.505 |
| **Postoperative Month 6** | 54.43±23.19 | 57.83±23.51 | 62.28±25.85 | 0.687 |
| **Postoperative Month 12** | 52.15±25.02 | 57.69±23.19 | 63.42±23.68 | 0.501 |
